# Bioinformatics Analysis Reveals Abundant Short Alpha-Helices as a Common Structural Feature of Oomycete RxLR Effector Proteins

**DOI:** 10.1371/journal.pone.0135240

**Published:** 2015-08-07

**Authors:** Wenwu Ye, Yang Wang, Yuanchao Wang

**Affiliations:** Department of Plant Pathology, Nanjing Agricultural University, Nanjing, China; James Hutton Institute, UNITED KINGDOM

## Abstract

RxLR effectors represent one of the largest and most diverse effector families in oomycete plant pathogens. These effectors have attracted enormous attention since they can be delivered inside the plant cell and manipulates host immunity. With the exceptions of a signal peptide and the following RxLR-dEER and C-terminal W/Y/L motifs identified from the sequences themselves, nearly no functional domains have been found. Recently, protein structures of several RxLRs were revealed to comprise alpha-helical bundle repeats. However, approximately half of all RxLRs lack obvious W/Y/L motifs, which are associated with helical structures. In this study, secondary structure prediction of the putative RxLR proteins was performed. We found that the C-terminus of the majority of these RxLR proteins, irrespective of the presence of W/Y/L motifs, contains abundant short alpha-helices. Since a large-scale experimental determination of protein structures has been difficult to date, results of the current study extend our understanding on the oomycete RxLR effectors in protein secondary structures from individual members to the entire family. Moreover, we identified less alpha-helix-rich proteins from secretomes of several oomycete and fungal organisms in which RxLRs have not been identified, providing additional evidence that these organisms are unlikely to harbor RxLR-like proteins. Therefore, these results provide additional information that will aid further studies on the evolution and functional mechanisms of RxLR effectors.

## Introduction

Within the context of host-pathogen interactions, ‘effectors’ represent a class of molecules and proteins secreted by pathogens to manipulate host cell processes. Effectors are classified into two types, namely apoplastic effectors and cytoplasmic effectors, that target distinct sites in host plants during pathogen infection [1]. One of the most typical and best-studied classes of cytoplasmic effectors in oomycetes is those that harbor an RxLR motif, which can be delivered inside plant cells during infection [2]. On one hand, RxLR effectors function as virulence factors since they can modulate cellular processes, mainly by suppressing plant immunity [3]; on the other hand, RxLR effectors function as avirulence factors once recognized by plant immune receptors. To date, 18 oomycete avirulence genes encoding RxLR effectors have been cloned [4]. However, host targets for the biological functions of RxLR effectors and their virulence- or avirulence-associated interaction networks remain largely unknown.

RxLR proteins are defined as modular. Typically, they carry a signal peptide followed by a conserved RxLR motif (arginine, anything, leucine, arginine); and many, but not all, carry a more variable second motif, termed dEER (aspartate, less well-conserved, glutamate, glutamate, arginine) at varying distances from the C-terminus to the RxLR motif. Approximately half of the encoded RxLR effectors are reported to contain additional repeating blocks in the C-terminus made up of a consecutive combination of W, Y and L motifs [5]. Moreover, most RxLR protein sequences are highly diverse, even among related species, and lack similarity to any other known proteins.

The family of RxLR effectors comprises approximately 350 to 550 members in each genome-sequenced species of the genus *Phytophthora*, which includes many devastating hemibiotrophic plant pathogens [5–7]. However, the number of RxLR effector genes is dramatically reduced to only 134 members in the biotrophic pathogen *Hyaloperonospora arabidopsidis* genome [8]. Reduced numbers of putative RxLR or RxLR-like effectors are also found in the genomes of several biotrophic pathogens, including *Pseudoperonospora cubensis* (61) [9], *Bremia lactucae* (78) [10], *Albugo candida* (26) [11], and *Albugo laibachii* (25) [12]. Thus far, genomes of seven necrotrophic *Pythium* species have been sequenced, but no RxLR effectors have been found [13–14]. Beyond the peronosporales, no evident RxLR gene has been identified, e.g., in *Saprolegnia parasitica*, an animal oomycete pathogen [15]. Moreover, in *Aphanomyces euteiches* [16], a biotrophic oomycete plant pathogen, although three predicted unigenes may encode RxLR-dEER or -like motifs, there is no further evidence to verify this prediction.

Defining the protein structure is an important molecular strategy to understand the functions of effectors and dissect the underlying molecular interaction mechanism. To date, crystal structures or nuclear magnetic resonance (NMR) data have been generated for six RxLR effectors, including *H*. *arabidopsidis* ATR1 [17] and ATR13 [18], *Phytophthora capsici* Avr3a4 [19] and Avr3a11 [20], *Phytophthora infestans* PexRD2 (or PiRD2) [20], and *Phytophthora sojae* Avh5 [21]. The structures of all of these proteins, with the exception of HaATR13, comprise multiple alpha-helical folds. HaATR13 has a distinct structure, consisting of three helices and a disordered loop at the C-terminus. The folds comprising three helices of *PcAvr3a4*, *PcAvr3a11*, *PsAvh5*, *PexRD2*, and *HaATR1* span their conserved C-terminal W and Y motifs. These are also defined as WY motifs, in which the highly conserved tryptophan and tyrosine residues contact each other to form the hydrophobic core of the fold [4, 20, 22]. The WY motif is believed to form a flexible scaffold that supports rapid changes in the primary sequence and structural architecture of RxLR effectors driven by the host-pathogen co-evolutionary conflict [22].

However, based on a sequence search, the WY motif is predicted to exist in only 44% of the annotated RxLR candidates in *P*. *infestans*, *Phytophthora ramorum* and *P*. *sojae*, and 26% in *H*. *arabidopsidis* [20, 22]. Hence, it will be interesting to analyze the protein secondary structures of currently known RxLR candidates, especially those that do not contain the W/Y/L motif. In the study described herein, a large-scale prediction of protein secondary structure and surface accessibility was performed, which revealed common structural features among sequence-diverse oomycete RxLR effectors. The results extend our understanding of RxLR effectors in protein secondary structures from individual or partial members to the entire family. Although large-scale determination of protein structure has been difficult thus far, the work reported herein provides new insights into the highly important oomycete effector family.

## Materials and Methods

### Collection of protein sequences and structural data

Protein sequences of *P*. *sojae* (v1.1), *P*. *ramorum* (v1.1) and *P*. *capsici* (v1.0) were obtained from the DOE Joint Genome Institute (JGI) database (genome.jgi.doe.gov); *P*. *infestans* (release of 6/15/2009), *F*. *graminearum* (FG3), *F*. *oxysporum* (4287), *Verticillium dahlia* (VdLs.17), and *M*. *oryzae* (MG8) were obtained from the Broad Institute database (www.broadinstitute.org); *H*. *arabidopsidis* (v8.3) was obtained from eumicrobedb.org; *B*. *lactucae* was obtained from testweb.science.uu.nl/pmi/data/bremia; and *Py*. *ultimum* (DAOM BR144; pug1) was obtained from the *Pythium* Genome Database (pythium.plantbiology.msu.edu). RxLR sequences were collected from published data, as mentioned in the ‘Introduction’. The determined protein secondary structures of RxLR proteins were obtained from the Protein Data Bank (PDB, www.rcsb.org).

### Prediction of protein secondary structure

Protein secondary structures were predicted using NetSurfP 1.1 (www.cbs.dtu.dk/services/NetSurfP; also predicts protein surface accessibility) and Proteus 2.0 (www.proteus2.ca/proteus2). For NetSurfP 1.1, the protein secondary structure class (alpha-helix, beta-strand and coil) predicted to have the greatest possibility was considered as the result. Surface accessibility results were obtained directly from the default output. For Proteus 2.0, ‘Eukaryote’ was selected as the organism type. Calculation of structural content and data analyses were performed using Microsoft Office Excel 2007.

### Identification of putative secreted proteins

Proteins were submitted to SignalP 3.0 (www.cbs.dtu.dk/services/SignalP-3.0), TargetP 1.1 (www.cbs.dtu.dk/services/TargetP) and TMHMM 2.0 (www.cbs.dtu.dk/services/TMHMM) to predict signal peptides, subcellular location and transmembrane helices, respectively. The defined secretome should simultaneously meet a SignalP HMM score > 0.9, subcellular localization as secreted (TargetP; default parameters), and no transmembrane domain after the signal peptide cleavage site (TMHMM; default parameters).

### Analyses of W/Y/L motifs

The W/Y/L motifs were predicted using the HMMER v3.0 package [23] with ‘0’ as the cut-off domain score. To build HMM models, alignments of W/Y/L motifs were obtained from the authors of previously published work [5]. Sequences were aligned using MUSCLE [24] with the FASTA and/or CLUSTALW output format. Weblogos were generated using Weblogo 3 (weblogo.threeplusone.com/create.cgi).

When comparing the proportions of alpha-helices among different regions of the RxLR proteins, the N-terminal signal peptide region referred to the N-terminal 25 amino acid (aa) peptide, the RxLR-dEER region referred to the peptide from the end of the signal peptide to the end of the RxLR-dEER motif, and the effector domain region referred to the remaining peptide. Proteins were discarded if the sequences corresponding to the abovementioned regions were shorter than 20 aa. For other analyses, the proportion of alpha-helices was calculated for the full-length proteins.

The W/Y/L-like regions were identified using BLASTP (version: 2.2.18-ia32-win32) [25] alignments against the identified W/Y/L motifs using ‘1’ as the e-value cut-off. In W/Y/L-like regions, the amino acids corresponding to the conserved sites of the W/Y/L motifs were manually identified. Amino acids I (Ile), L (Leu), F (Phe), V (Val), M (Met), W (Trp), and C (Cys) were classified into the ‘very hydrophobic’ group; Y (Tyr), A (Ala), T (Thr), G (Gly), S (Ser), H (His), and P (Pro) were classified into the ‘less hydrophobic’ group; K (Lys) and R (Arg) were classified into the ‘part hydrophobic’ group; and D (Asp), E (Glu), N (Asn), and Q (Gln) were classified into the ‘hydrophilic’ group [26].

### Identification of alpha-helix-rich proteins

Candidate alpha-helix-rich proteins should originate from the identified secretomes and meet three criteria: 40% < alpha-helix% < 70%, beta-strand% < 10%, and 25% < random coil < 50%. Candidate proteins were further categorized as RxLR proteins, function-annotated proteins, and others, respectively. The function-annotated proteins represent the non-RxLR proteins belonging to a superfamily after querying the Batch CD-Search tool in NCBI (www.ncbi.nlm.nih.gov/cdd) using default parameters.

## Results

### The majority of RxLR proteins contain abundant alpha-helices

**Fig 1:**
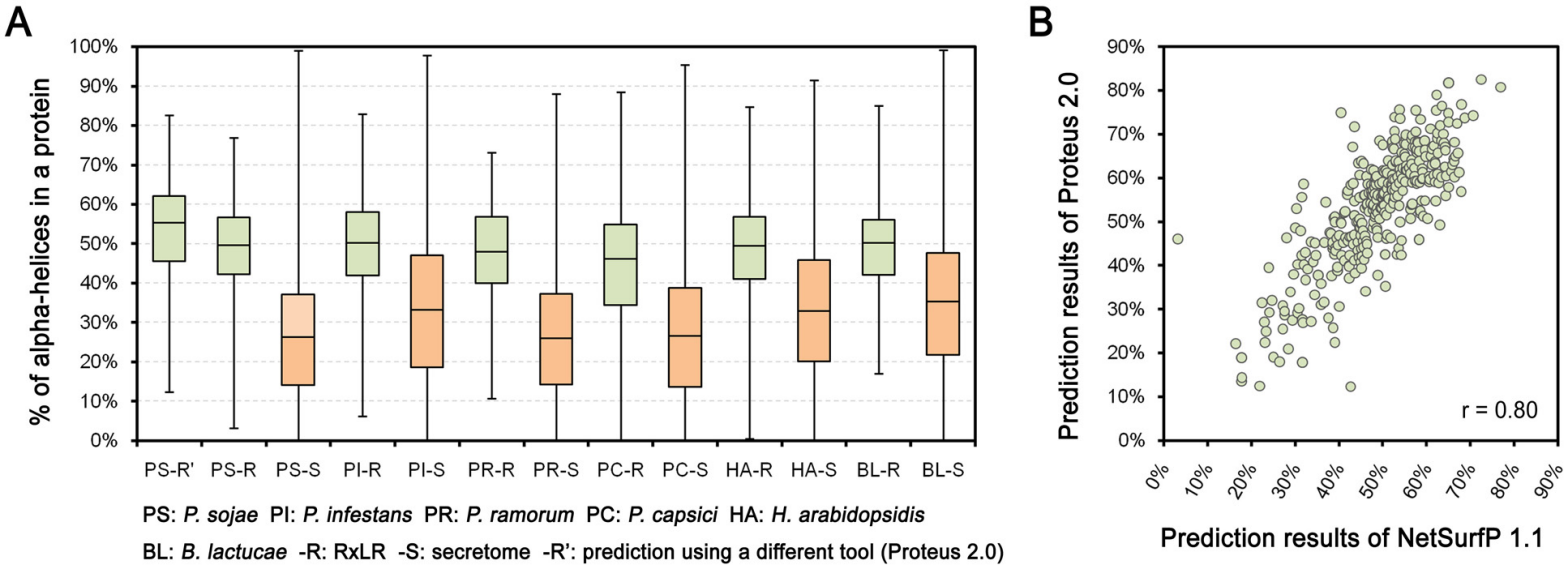
Comparison of the proportions of alpha-helices between RxLR proteins and secretomes among species. (A) Boxplots indicate the proportions of alpha-helices in different protein sets. The bottom and top of the box represent the first and third quartiles, and the band inside the box represents the median. The whisker ends represent the minimum and maximum of all data. (B) The scatter diagram indicates the correlation between the results of two protein secondary structural prediction tools, NetSurfP 1.1 and Proteus 2.0. ‘r’ refers to the Pearson correlation coefficient.

To examine the structural features of RxLR effector proteins, NetSurfP 1.1 [27] was used for protein secondary structure prediction. We found that the majority of RxLR proteins were predicted to fold abundant alpha-helices in *P*. *sojae* (S1 Fig). The median proportion of alpha-helices in each RxLR was 49%, nearly twofold higher than that of the secretome (27%) (Fig 1A, Table A in S1 File). Similar results were obtained in *P*. *infestans* (49% *vs*. 33%), *P*. *ramorum* (48% *vs*. 27%), *P*. *capsici* (45% *vs*. 28%), *H*. *arabidopsidis* (48% *vs*. 33%), and *B*. *lactucae* (48% *vs*. 36%) (Fig 1A, Table A in S1 File). To evaluate prediction reliability, protein structure prediction was also performed using Proteus 2.0 [28]. Predicted alpha-helix proportions for *P*. *sojae* RxLRs from both tools exhibited a high positive Pearson’s correlation (r = 0.80) (Fig 1B, Table A in S1 File). The median proportion of alpha-helices in each RxLR predicted from Proteus 2.0 was 55%, which is even higher than that from NetSurfP 1.1 (49%) (Fig 1A). In addition, the prediction results from NetSurfP 1.1 and Proteus 2.0 showed overall identities of 73.4% and 73.5%, respectively, against the assayed results of the five RxLRs whose structures were determined previously (S2 Fig). Therefore, we conclude that the prediction tools demonstrated a normal performance, and the majority of RxLR proteins contain abundant alpha-helices.

### Abundant alpha-helices are distributed in partial regions of RxLR proteins

**Fig 2:**
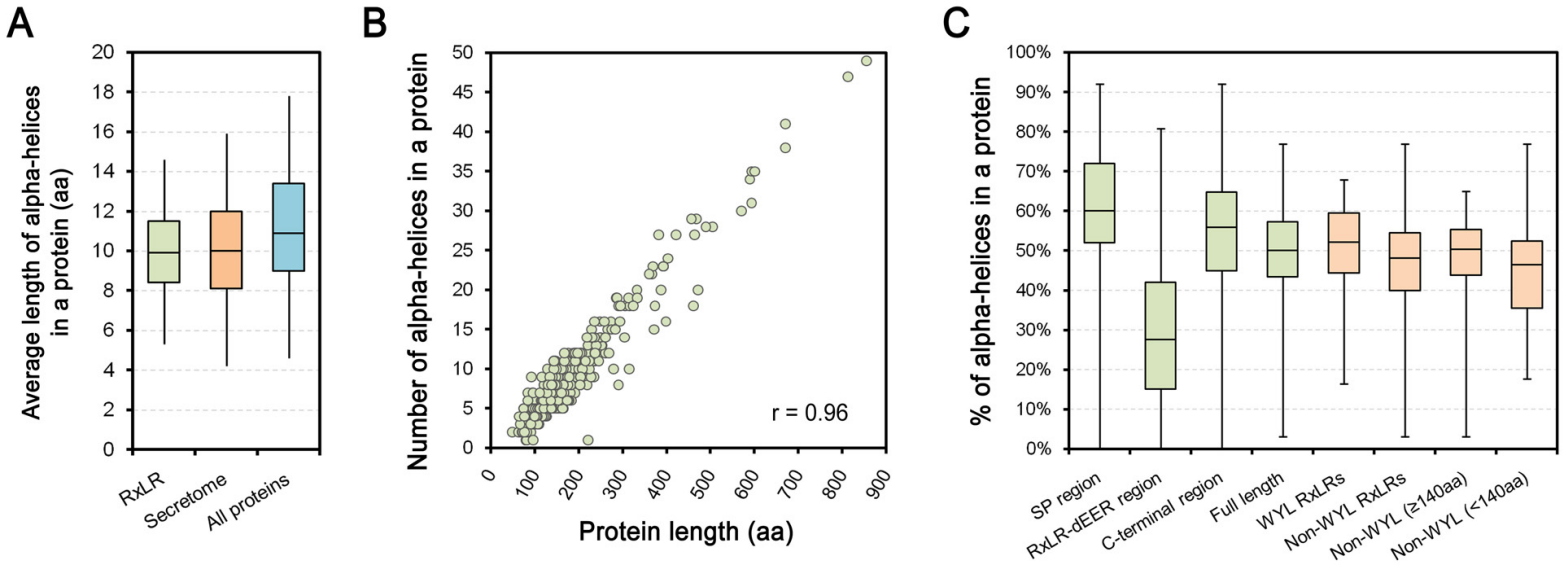
Characteristics of predicted alpha-helices of *P*. *sojae* RxLR proteins. (A) Boxplots indicate the Mean Lengths of Alpha-helices (MLAs) in different protein sets. The bottom and top of the box represent the first (Q1) and third (Q3) quartiles, and the band inside the box represents the median. The whisker ends reach Q3+1.5IQR and Q1-1.5IQR (IQR = Q3-Q1), respectively. (B) The scatter diagram describes the correlation between the numbers of alpha-helices in each protein and their corresponding lengths. ‘r’ refers to the Pearson correlation coefficient. (C) The boxplots describe the proportions of alpha-helices in different regions or categories of RxLR proteins. The bottom and top represent the first and third quartiles, and the band inside the box represents the median. The whisker ends represent the minimum and maximum of all data.

Protein structural features of RxLR effectors were further characterized according to prediction results from NetSurfP 1.1 in *P*. *sojae*. We calculated the Mean Length of the Alpha-helices in each protein (MLA), and found that over half in MLA are 8–12 aa. The median MLAs among RxLR proteins were slightly shorter than whole genome proteins (9.9 aa *vs*. 10.9 aa), but similar to the secretome proteins (10.0 aa) (Fig 2A, Table B in S1 File). In addition, the total numbers of alpha-helices in each RxLR protein exhibited a strong correlation (r = 0.96) with protein length (Fig 2B).

However, the predicted alpha-helices were not uniformly distributed throughout the RxLR protein sequence. Upon comparing the N-terminal signal peptide (SP) regions, the following RxLR-dEER regions, the remaining C-terminal effector domain regions, and the full-length RxLR proteins (see ‘Materials and methods’), both the SP and C-terminal effector domain regions were predicted to contain high proportions of alpha-helices (60% and 56% in median, respectively). These values are slightly higher than those of the full-length proteins. In contrast, alpha-helices were more sparse in the RxLR-dEER region (30%) (Fig 2C, Table C in S1 File). Since the SP region is relatively short and contributes little to the full-length protein with regard to determining alpha-helix proportions, most RxLR proteins with abundant alpha-helices are associated with the C-terminal region, not the RxLR-dEER region.

### High conservation of C-terminal W/Y/L regions in protein secondary structure

**Fig 3:**
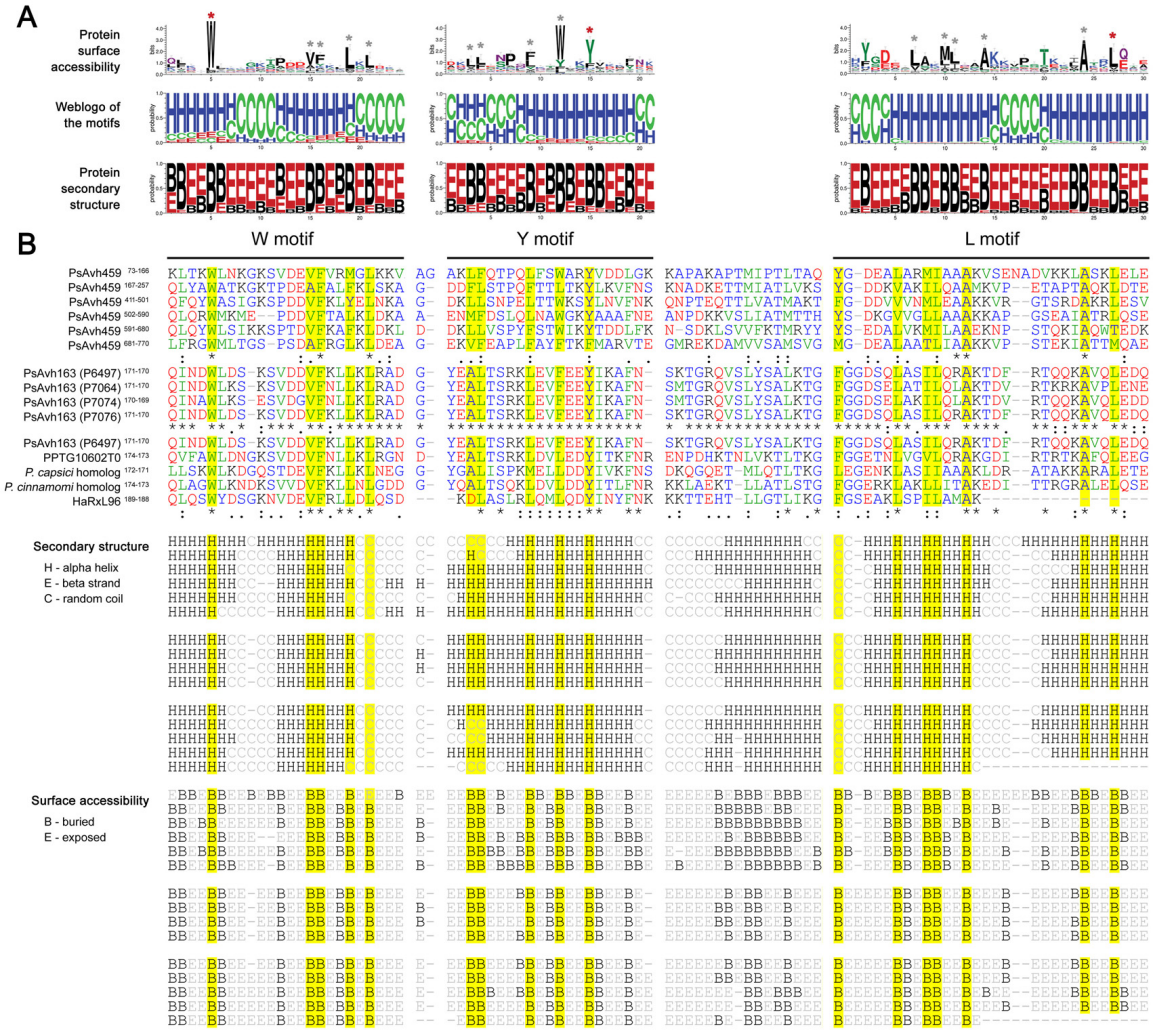
Sequence, protein secondary structure and surface accessibility alignments of W-Y-L regions. (A) Alignments of all *P*. *sojae* W/Y/L motifs. With respect to protein surface accessibility, ‘B’ and ‘E’ represent buried and exposed residues, respectively; and with respect to secondary structure, ‘H,’ ‘E’ and ‘C”‘ represent alpha helix, beta strand and random coil, respectively. Asterisks refer to the mentioned conserved sites within W/Y/L motifs. Red asterisks indicate the amino acids that give the W/Y/L motifs their names. (B) Representative alignments of W-Y-L regions within PsAvh459, or the same W-Y-L loci, but from different products of *PsAvh163* alleles, or from PsAvh163 and its orthologs. Yellow indicates conserved sites within the W/Y/L motifs, which correspond to those marked by grey asterisks in (A). Below the sequence alignment, ‘*’ indicates positions with a fully conserved residue, while ‘:’ and ‘.’ indicate strongly and less strongly conserved residues, respectively. Letters referring to very hydrophobic, less hydrophobic, part hydrophobic, and hydrophilic amino acids are colored in green, blue, black, and red, respectively.

With respect to the C-terminal sequences of RxLR proteins in *P*. *sojae* and other species, nearly no conventional “function-known” domain has been found, with the exception of PsAvr3b, which contains a Nudix hydrolase domain following partial conventional RxLR effector leading [29]. Based on our results, full-length PsAvr3b was predicted to contain fewer alpha-helices (NetSurfP 1.1, 38%; Proteus 2.0, 28%), further suggesting that PsAvr3b is a minor case within the RxLR family. In addition, several RxLR protein sequences have been determined to contain repeats of W, Y and L motifs, or WY motifs, which were identified from the sequences of RxLRs [5, 20, 22]. In this study, the RxLRs that contained and did not contain W/Y/L motif(s) were termed ‘WYL RxLRs’ and ‘non-WYL RxLRs,’ respectively (Table C in S1 File). Respective alignments of the W, Y and L motifs showed that only a few sites are conserved. The amino acids at these sites included Trp (W), Val (V), Phe (F), Leu (L), Tyr (Y), and Ala (A), and were classified into the ‘very hydrophobic’ or ‘less hydrophobic’ group (see ‘Materials and methods’) [26]. In contrast, sequences at other sites were highly diverse (Fig 3A and 3B).

However, the predicted protein secondary structures of W/Y/L motifs are considerably more conserved. Generally, the motifs were predicted to fold one to two alpha helix(s), each being 8–12 aa in length. Most of the abovementioned conserved and hydrophobic amino acids were predicted to be buried (lower protein surface accessibility) and within the alpha-helices (Fig 3A). For example, the different W-Y-L regions within PsAvh459, or the same W-Y-L loci but from different products of *PsAvh163* alleles, or from PsAvh163 and its orthologs, all exhibited high conservation in protein secondary structure, as mentioned above (Fig 3B). In addition, interspaces between those Y- and L motifs, each including 16 amino acids, also displayed protein secondary structure conservation, while W and Y, and L and W motifs were always closely connected (Fig 3B). Thus, from these results, we conclude that the C-terminal W-Y-L regions of RxLRs are highly conserved in terms of protein secondary structure.

### C-terminal sequence variation is associated with protein structure

**Fig 4:**
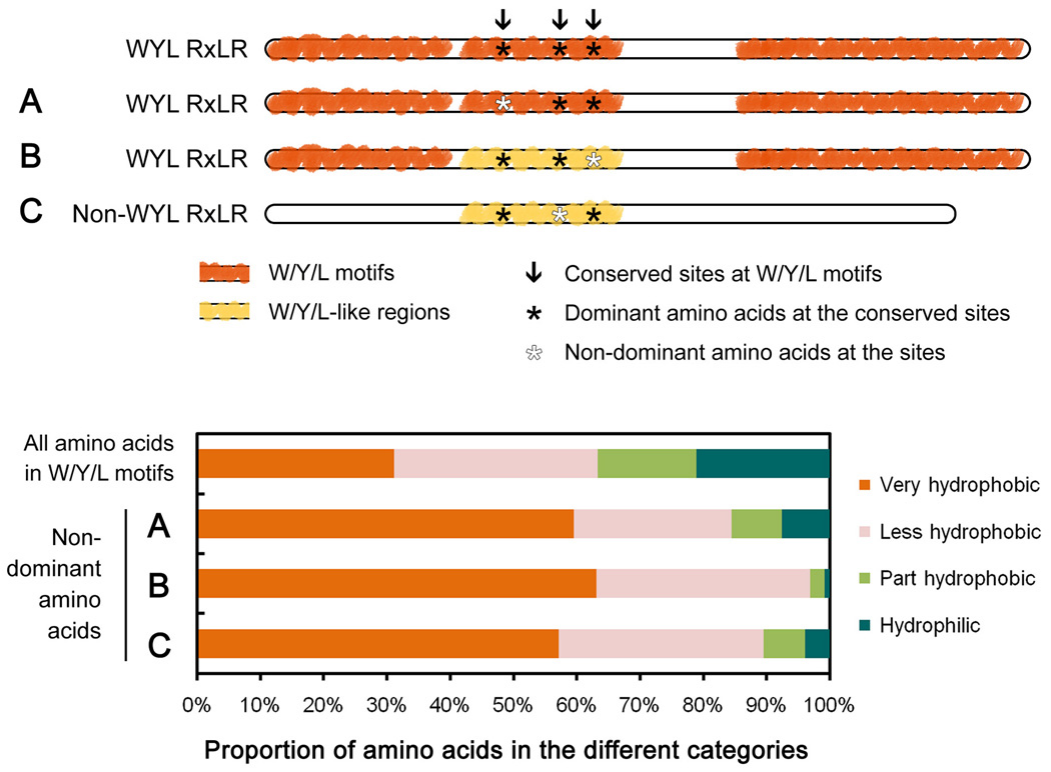
Characteristics of amino acids at the conserved W/Y/L motif sites. With regards to the non-dominant amino acids corresponding to the conserved W/Y/L motif sites, (A) indicates the W/Y/L motifs, (B) indicates the W/Y/L-like regions of WYL RxLRs, and (C) indicates the W/Y/L-like regions of non-WYL RxLRs. The features of all amino acids in the W/Y/L motifs are also described. Amino acids I (Ile), L (Leu), F (Phe), V (Val), M (Met), W (Trp), and C (Cys) belong to the ‘very hydrophobic’ group; Y (Tyr), A (Ala), T (Thr), G (Gly), S (Ser), H (His), and P (Pro) belong to the ‘less hydrophobic’ group; K (Lys) and R (Arg) belong to the ‘part hydrophobic’ group; and D (Asp), E (Glu), N (Asn), and Q (Gln) belong to the ‘hydrophilic’ group.

Although the hydrophobic amino acids in certain sites of the W/Y/L motifs are conserved (Fig 3A and 3B), we also characterized cases in which amino acids at those sites were not dominant. We found the frequency of very hydrophobic amino acids from the non-dominant amino acids corresponding to conserved sites within the W/Y/L motifs (60%) was greater than in full-length *P*. *sojae* W/Y/L motifs (31%) (Fig 4A). In addition, since variation at the conserved sites and/or loss, gain or recombination of the motif(s) [30] may result in negative prediction of the W/Y/L motifs in the previous HMM search, we identified several W/Y/L-like sequences from non-W/Y/L-motif regions of the WYL RxLRs based on a Blastp search using ‘1’ as a relax cut-off of e-value. Among the 130 identified non-dominant amino acids corresponding to conserved sites within the W/Y/L motifs, 63% were very hydrophobic (Fig 4B, Table C in S1 File: column Z).

Furthermore, we found no clear difference between the non-WYL RxLR and WYL RxLR proteins (S1 Fig, Table C in S1 File) in proportions of alpha-helices, although the median value of non-WYL RxLRs was slightly lower (48% *vs*. 52%, respectively) (Fig 2C). This value increased to 50% after excluding a number of short non-WYL RxLRs (full-length < 140 aa) (Fig 2C), which were inferred to be too short to contain at least one complete W, Y or L motif (S2 Fig). On the basis of a similar Blastp search, we further identified 31 mutated and/or partial W/Y/L-motif-like regions from 26 non-WYL RxLRs (WYL-like RxLRs). As expected from the results described above, a high proportion (57%) of the amino acids at the conserved sites within the W/Y/L motifs was still hydrophobic following native mutation (Fig 4C, Table C in S1 File: column Z).

Upon comparison with P6497, we found DNA polymorphisms in three other representative isolates from *P*. *sojae* (P7064, P7074 and/or P7076) [3], located at 629 nucleotides corresponding to 539 codons within the WYL- and WYL-like RxLR proteins. Only 22 corresponded to conserved W/Y/L motif sites; 14 of which resulted in non-synonymous substitutions, and six (43%) that remained very hydrophobic (Table C in S1 File: column AB). These results indicate that most amino acids of RxLR proteins corresponding to conserved W/Y/L motif sites are maintained as hydrophobic, which may be associated with the conserved helical scaffolds of RxLR proteins.

### RxLR effectors with abundant alpha-helices differ from other effector family members

**Fig 5:**
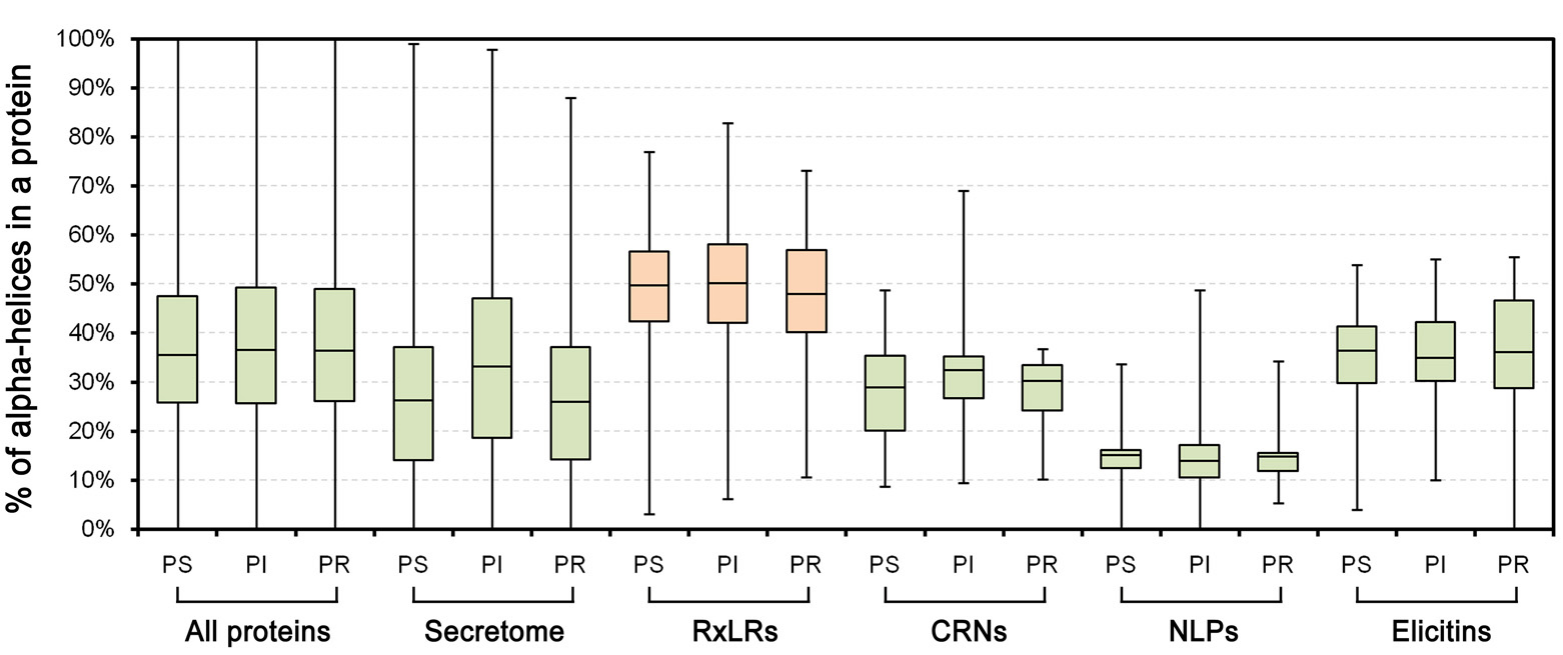
Comparison of alpha-helix proportions among effector families. Boxplots show the proportions of alpha-helices in different protein sets. The bottom and top of the box represent the first and third quartiles, and the band inside the box represents the median. The whisker ends represent the minimum and maximum of all data.

**Fig 6:**
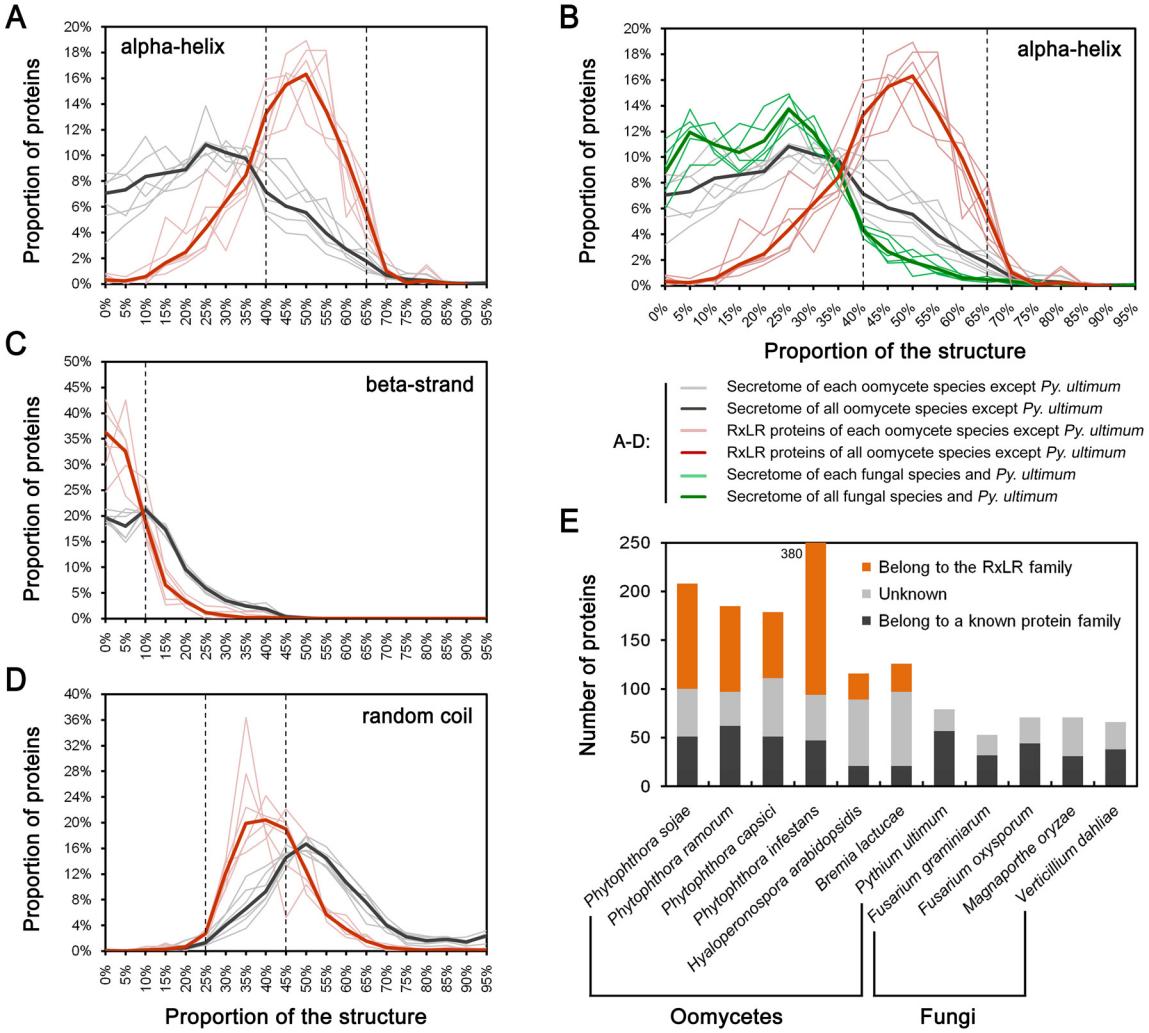
Comparison of alpha-helix-rich proteins in terms of proportions and numbers. (A-D) Proteins with different proportions of predicted secondary structures, including alpha-helices, beta-strands and random coils. Dotted lines indicate the critical point for preliminary identification of alpha-helix-rich candidates. With respect to the proportional values along the *x*-axis, e.g., 95% indicates a range equal or higher than 95% and smaller than 100% (95%+5%). (E) Numbers of candidate alpha-helix-rich secreted proteins identified from seven oomycete species and four fungal species.

We further compared the protein secondary structural features of RxLRs with other *Phytophthora* proteins, especially those from other effector families. We found that the whole-genome proteins contained a higher proportion of alpha-helices than those predicted based on the secretome (median, 36% *vs*. 26%); however, the RxLR proteins exhibited greater values than the whole-genome proteins (49% *vs*. 36%) (Figs 5 and 6A). We also analyzed other *Phytophthora* multigene effector families (i.e., CRN, NLP and elicitin) (Table D), and found that their median proportions of alpha-helices were diverse but much lower than the RxLR family and even the whole-genome proteins (i.e., NLPs (15%) < secretome (27%) < CRNs (29%) < elicitins (36%) < all proteins (36%) < RxLRs (49%)) in *P*. *sojae* (Fig 5). Parallel analyses were performed in *P*. *ramorum* and *P*. *infestans*, and similar results were obtained (Fig 5, Table D in S1 File). Therefore, the different protein secondary structural features revealed a clear distinction of the RxLR family among the *Phytophthora* proteins.

Upon comparing the secretomes of six RxLR-containing species, including oomycete *P*. *sojae*, *P*. *ramorum*, *P*. *capsici*, *P*. *infestans*, *H*. *arabidopsidis*, and *B*. *lactucae*, and the other five species, including oomycete *Py*. *ultimum* and fungal *Fusarium graminearum*, *Fusarium oxysporum*, *Magnaporthe oryzae*, and *Verticillium dahlia*, we found that all species in the first group had markedly higher proportions of alpha-helix-rich proteins than those in the latter group (Fig 6B: black lines *vs*. green lines). The increased number of alpha-helix-rich proteins in RxLR-containing species may be due to the existence of the RxLR proteins (Fig 6B: red lines). To validate this hypothesis, all of the abovementioned secretomes were scanned using the following three criteria: 40% < alpha-helix% < 70%, beta-strand% < 10%, and 25% < random coil <50%. These criteria were defined according to critical points along the distribution curves between RxLR proteins and their respective secretomes (Fig 6A–6D). More candidates were obtained from the six RxLR-containing species, especially those of *Phytophthora*. However, after excluding those belonging to the RxLR family, the number of candidates in each species was almost equal, although the other five species exhibited fewer candidates. Thus, the expansion of RxLR proteins may be a major reason for the higher proportion of alpha-helix-rich proteins in the secretomes of oomycete plant pathogens.

## Discussion

In the study described herein, we determined that the presence of abundant short alpha-helices is a common protein secondary structural feature of the RxLR effector family, based on bioinformatics analysis. This common feature is consistent with the results of previous structural studies on individual RxLR proteins, including PcAvr3a4, PcAvr3a11, PsAvh5, PexRD2, HaATR1, and HaATR13, the C-termini of which were determined to contain multiple alpha-helices [4, 19–20, 22]. Furthermore, the prediction tools utilized herein achieved over 73% accuracy when comparing the five available RxLR structures. In addition, we found that the RxLR-dEER region contains poor alpha-helices, consistent with previous results in which the N-terminal regions, including the RxLR-dEER region, are generally disordered [19–20]. Hence, while the protein secondary structure data were predicted, our preliminary results on RxLR protein structures have proven useful.

In line with the previous reports, we found only approximately half of the encoded RxLR effectors contain C-terminal W/Y/L or WY motifs [5, 20, 22]. However, the majority of RxLR proteins, irrespective of the presence of W/Y/L motifs, share abundant short alpha-helices in their secondary structures. As expected, under intense host defense pressure due to the ‘arms-race’ between plants and pathogens, the RxLR effector genes are evolving rapidly to escape host detection and adopt diverse virulence functions [5–6]. Their evolutionary paths have been found to accompany frequent loss or gain of their repeated effector domains, recombination or gene conversion among paralogs and selection of point mutations [30]. Thus on the one hand, the rapid variation among sequences may lead to the un-reorganization of the artificially defined canonical W/Y/L motifs in non-WYL RxLRs or non-W/Y/L regions of the WYL RxLRs.

On the other hand, although the RxLR protein sequences are highly diverse, we found the C-terminal amino acids corresponding to the suggested helical ‘cores’ tend to be conserved or hydrophobic following native mutation. In addition, few cases of recombination between RxLR genes and non-RxLR genes (which may have different structural feature) are known, the exception being *PsAvr3b* [29]. It is well-known that an approximately 30% sequence identity is sufficient to support identical or similar tertiary structures [31]. Thus, the evidence presented herein supports that non-WYL RxLRs maintain similar structural features.

To date, with the exception of certain species of oomycete plant pathogens known to encode RxLR effectors, no significant RxLR homolog has been identified in other oomycete [13, 15] or fungal organisms. In this study, we found that six previously analyzed RxLR-containing species, especially those of *Phytophthora*, contain a greater number of alpha-helix-rich protein candidates. In contrast, the number of identified non-RxLR and alpha-helix-rich proteins is similar among all studied oomycete and fungal species. Although these results do not demonstrate the absence of RxLR proteins in other oomycete and fungal organisms, they provide additional evidence that these organisms are unlikely to harbor RxLR- or RxLR-like proteins. Even though being existent in other oomycete and fungal organisms, the RxLR-like family in protein number is unlikely to be expanded. In addition, since the abundance of alpha-helices in the RxLR effector family differs from that in other effector families, genes in the RxLR family are likely independently evolving and dramatically expanding in certain oomycete genomes.

In conclusion, although a large-scale experimental determination of protein structures has been difficult to date, the results of the study described herein extend our understanding of important oomycete RxLR effectors in protein secondary structures from individual or partial members to the entire family. The majority of RxLR proteins share a common feature, a helical protein scaffold, which is beyond the previous understanding that be associated with W/Y/L motifs. Thus, these results provide additional information that will aid further studies on the evolution and functional mechanisms of RxLR effectors.

## Supporting Information

S1 Fig Diagrams of predicted W/Y/L motifs and alpha helices in all *P*. *sojae* RxLR proteins. The full-length proteins are indicated by grey lines. W, Y and L motifs, and predicted alpha helices are indicated by red, yellow, blue, and black blocks, respectively. Lines and blocks are proportional to sequence length.
(JPG)

S2 Fig Comparison of determined and predicted secondary structures of RxLRs. The determined protein secondary structures of five RxLR proteins were obtained from the Protein Data Bank (PDB; www.rcsb.org); the PDB IDs are displayed in parentheses. Values on the left of NetSurP and PROTEUS2 represent the ‘predicted’ against the ‘determined’ results.
(JPG)

S3 Fig Relationship between protein length and number of W/Y/L motifs. Scatter diagram of the relationship between protein length and number of W/Y/L motifs among *P*. *sojae* WYL RxLRs. A high Pearson’s correlation coefficient was obtained (r = 0.96). According to the inferred linear equation, y = 0.032x-3.484 (y, motif number; x, protein length), we speculate that proteins of less than 140 aa (y < 1) may be too short to encode a peptide containing W, Y, or L motifs.
(JPG)

S1 File Table A. Predicted protein secondary structures of RxLRs and secretomes from different oomycetes. Table B. Mean length of predicted alpha-helices. Table C. Prediction data and analyzed results from *P*. *sojae* RxLRs. Table D. Predicted protein secondary structures of other *Phytophthora* effectors.
(XLSX)
