# Supplementary figures and images for: Bioinformatics Analysis Reveals Abundant Short Alpha-Helices as a Common Structural Feature of Oomycete RxLR Effector Proteins
Source: PLoS One. 2015 Aug 7;10(8):e0135240. doi: 10.1371/journal.pone.0135240 (PMC4529148; doi:10.1371/journal.pone.0135240)

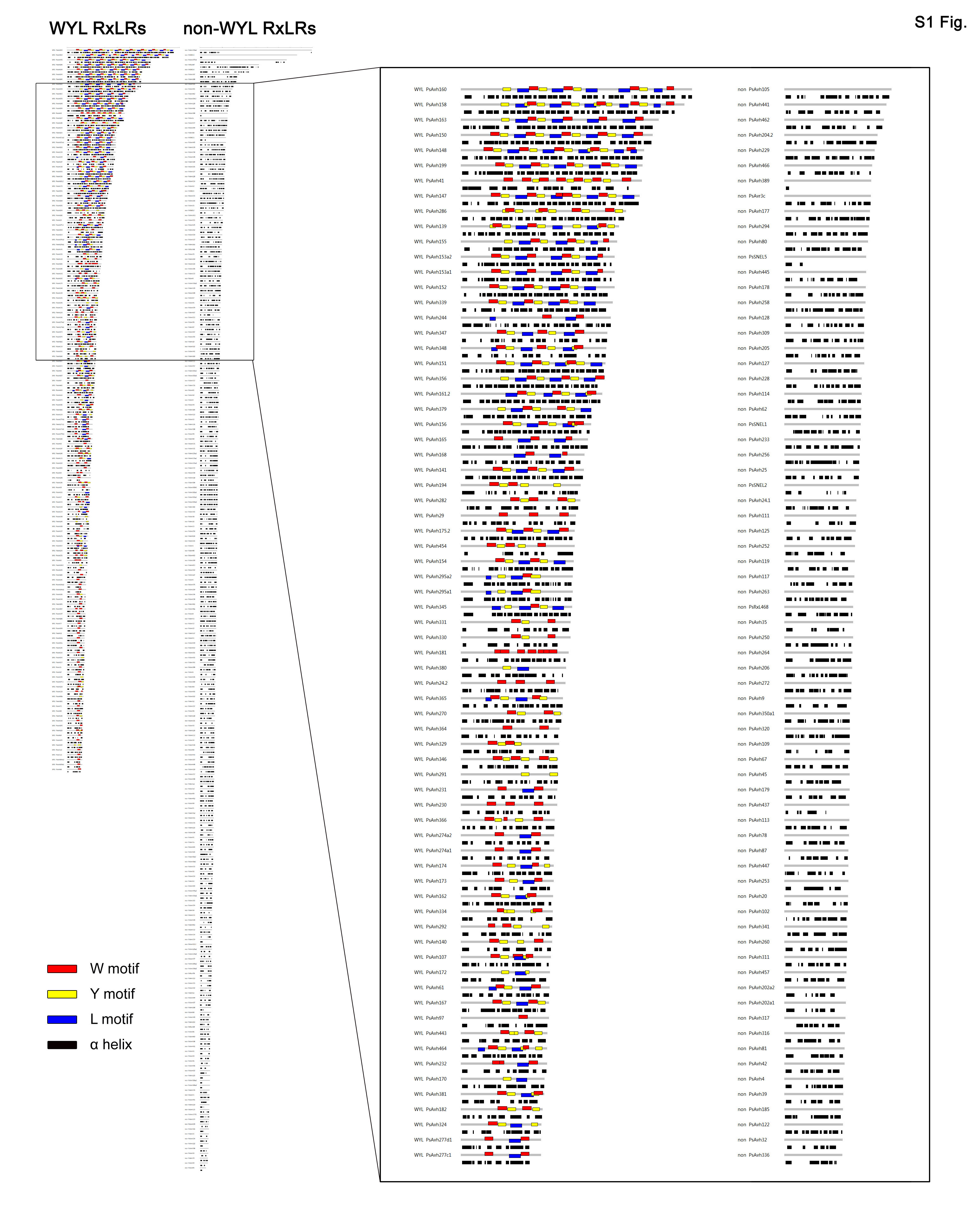

Supplement: S1 Fig — The full-length proteins are indicated by grey lines. W, Y and L motifs, and predicted alpha helices are indicated by red, yellow, blue, and black blocks, respectively. Lines and blocks are proportional to sequence length. (JPG) [file pone.0135240.s001.jpg]

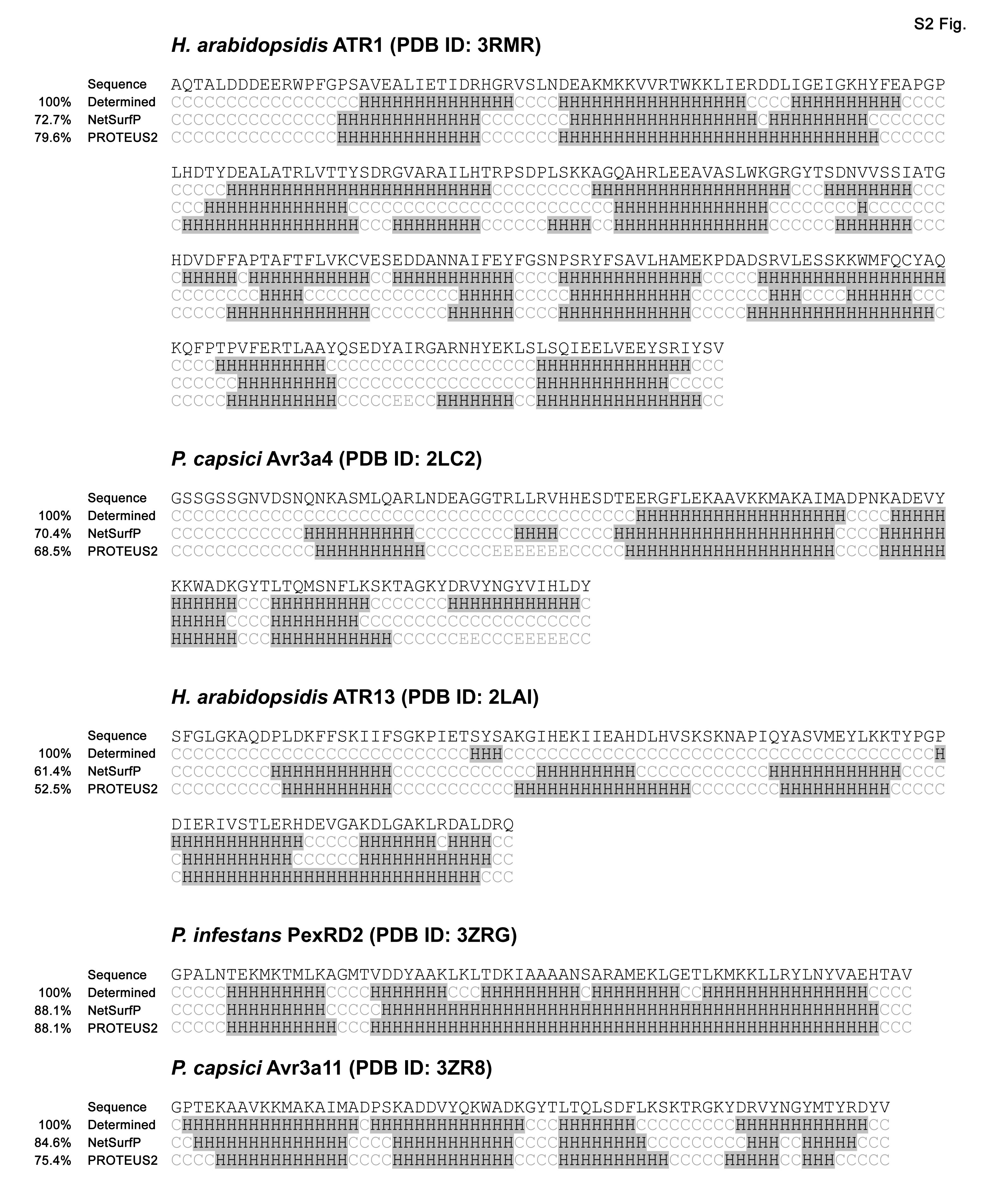

Supplement: S2 Fig — The determined protein secondary structures of five RxLR proteins were obtained from the Protein Data Bank (PDB; www.rcsb.org); the PDB IDs are displayed in parentheses. Values on the left of NetSurP and PROTEUS2 represent the ‘predicted’ against the ‘determined’ results. (JPG) [file pone.0135240.s002.jpg]

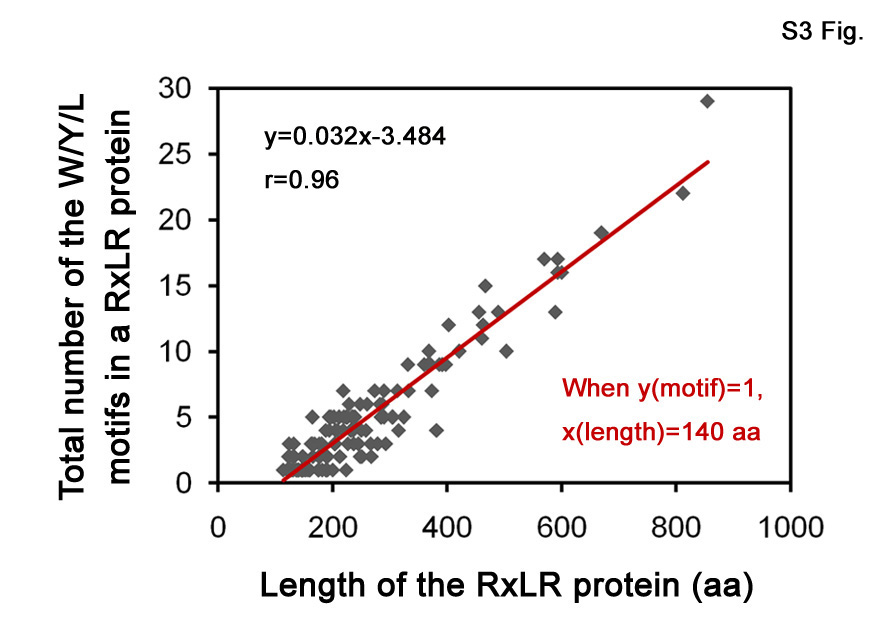

Supplement: S3 Fig — Scatter diagram of the relationship between protein length and number of W/Y/L motifs among P. sojae WYL RxLRs. A high Pearson’s correlation coefficient was obtained (r = 0.96). According to the inferred linear equation, y = 0.032x-3.484 (y, motif number; x, protein length), we speculate that proteins of less than 140 aa (y < 1) may be too short to encode a peptide containing W, Y, or L motifs. (JPG) [file pone.0135240.s003.jpg]
